# Lower lid entropion secondary to treatment with alpha-1a receptor antagonist: a case report

**DOI:** 10.1186/1752-1947-4-77

**Published:** 2010-03-02

**Authors:** Salman Waqar, Peter Simcock

**Affiliations:** 1 West of England Eye Unit, Royal Devon and Exeter NHS Hospital, Barrack Road, Exeter, Devon, EX2 5DS, UK

## Abstract

**Introduction:**

The use of alpha-1a receptor antagonists (tamsulosin) is widely accepted in the treatment of benign prostatic hypertrophy (BPH). It has previously been implicated as a causative agent in intra-operative floppy iris syndrome due to its effects on the smooth muscle. We report a case of lower lid entropion that may be related to a patient commencing treatment of tamsulosin.

**Case presentation:**

A 74-year-old Caucasian man was started on alpha 1-a receptor antagonist (Tamsulosin) treatment for benign prostatic hypertrophy. Eight days later, he presented to the ophthalmology unit with a right lower lid entropion which was successfully treated surgically with a Weiss procedure.

**Conclusion:**

We report a case of lower lid entropion that may be secondary to the recent use of an alpha-1a blocker (tamsulosin). This can be explained by considering the effect of autonomic blockade on alpha-1 receptors in the Muller's muscle on a patient that may already have an anatomical predisposition to entropion formation due to a further reduction in muscle tone.

## Introduction

The use of alpha-1a receptor antagonists (tamsulosin) is widely accepted in the treatment of benign prostatic hypertrophy (BPH). It has previously been implicated as a causative agent in the intra-operative floppy iris syndrome due to its effects on smooth muscle. We report a case of lower lid entropion that may be related to a patient commencing treatment with tamsulosin.

## Case presentation

A 74-year-old Caucasian man presented to the ophthalmology outpatient clinic with a five-day history of a heavy and sore right eye. His past ocular history included cataract surgery and left penetrating keratoplasty for Fuch's endothelial dystrophy. He was maintained on long term flurometholone eye drops once a day in his left eye. He also had been prescribed timolol eye gel (Nyogel) once a day to the left eye for ocular hypertension. His past medical history included paroxysmal atrial fibrillation and hypercholesterolemia. Other regular medications included oxazepam, clopidogrel, lanzoprazole, flecainide acetate and sildenafil. Eight days prior to presentation to the eye clinic, our patient consulted a urologist with complaints of frequency of micturition and had been started on tamsulosin (Flomaxtra XL) 0.4 mg once a day with good response.

On examination, he was found to have visual acuities of 6/9 with glasses improving to 6/6 with pinhole in both eyes. A right lower lid entropion was noted with moderate lid laxity. He subsequently underwent a right lower lid entropion repair (Weiss procedure) under local anaesthetic with good results.

## Discussion

Tamsulosin is the most commonly prescribed drug for the treatment of benign prostatic hyperplasia. It acts by selectively antagonising alpha-1a adrenergic receptors found in the bladder neck and prostate smooth muscle resulting in relaxation of the muscles and improvement of urinary flow. It has the therapeutic advantage of being uroselective and therefore has fewer cardiovascular side effects. A daily medication of 0.4 mg of tamsulosin has been found to be safe, well-tolerated and clinically effective in improving symptoms and urinary flow rate in patients with symptomatic BPH [1].

However, due to its effect on alpha-1a adrenergic receptors in iridial smooth muscle, it has also been documented to cause the intra-operative floppy iris syndrome (IFIS) [2]. It is believed that tamsulosin blocks the iris dilator muscle and this constant receptor blockade results in semi-permanent loss of muscle tone leading to a flaccid and floppy iris. However, no significant relationship has been found between the duration of tamsulosin intake and severity of IFIS [3].

The Muller's muscle is a smooth muscle that lies just deep to the orbital septum in both upper and lower eye lids. Its primary function is to assist in the retraction of both lids and is primarily innervated by alpha-2 adrenergic receptors although recent studies have also shown the presence of alpha-1 receptors [4]. This is of clinical significance in Horner's syndrome where the interruption of sympathetic supply to the muscle can result in ptosis and an elevation of the lower lid by as much as 1 mm [5]. Although not reported to be of benefit in entropion management, apraclonidine has shown improvement in lid function in Horner's syndrome. Apraclonidine is a weak alpha-1 agonist and a potent alpha 2 agonist. In Horner's syndrome, there is upregulation of alpha 1 receptors leading to denervation hypersensitivity. This, in turn, causes the observed lid retraction with apraclonidine [6,7]. Building on this knowledge and given the recent use of tamsulosinin our patient, we hypothesize that there was an alpha-1 blockade on the Muller's muscle in the right lower lid. This led to increased lower lid laxity, which may have been predisposed to the development of the entropion soon after commencing tamsulosin.

## Conclusion

We report a case of lower lid entropion that may be secondary to the recent use of an alpha-1a blocker (tamsulosin). This can be explained by considering the effect of autonomic blockade on alpha-1 receptors in the Muller's muscle on a patient that may already have an anatomical predisposition to entropion formation due to a further reduction in muscle tone.

## Consent

Written and informed consent was obtained from our patient for publication of this case report and accompanying images. A copy of the written consent is available for review by the Editor-in-Chief of this journal.

## Competing interests

The authors declare that they have no competing interests.

## Authors' contributions

SW and PS clinically diagnosed and managed our patient including the surgical intervention needed. Both authors were involved in writing the manuscript and approved the final version for submission.
